# Wild Poliovirus Type 1, Central African Republic

**DOI:** 10.3201/eid1109.050517

**Published:** 2005-09

**Authors:** Ionela Gouandjika-Vasilache, Jean Kipela, Regis Mbay Daba, Vicroire Mokwapi, Emmanuel Nambozuina, Joseph Cabore, Omer Pasi, Didier Menard

**Affiliations:** * Institut Pasteur de Bangui, Bangui, Central African Republic;; † World Health Organization, Bangui, Central African Republic;; ‡ Ministry of Health, Bangui, Central African Republic;; § World Health Organization, Yaounde, Cameroon;; ¶ Centers for Disease Control and Prevention, Atlanta, Georgia, USA

**Keywords:** wild poliovirus, type 1, outbreak, Central Africa, Central African Republic, letter

**To the Editor:** In this article we summarize the investigation and response to the reemergence of wild poliovirus (WPV) type 1 in the Central African Republic (CAR) in 2003. Since 2000, reported annual routine vaccination coverage with >3 doses of oral polio vaccine (OPV) has been very low in CAR (<50%); National Immunization Days have been conducted every year since 1996, except in 2002 (*1*).

From December 2003 to November 2004, the active acute flaccid paralysis surveillance system reported 112 cases of acute flaccid paralysis suspected to be poliomyelitis and 4 deaths (case-fatality ratio 4%). Fecal samples were collected and sent to the Institut Pasteur de Bangui. WPV type 1 (WPV1) was isolated in 30 cases (27%), vaccine polioviruses in 15 cases (5 type 1, 5 type 2, and 6 type 3) (13%), and nonpolio enteroviruses in 18 cases (16%). Epidemiologic investigations showed that 97% of patients with poliomyelitis received <3 doses of OPV and 93% of patients were <5 years of age. Isolates were sent to the National Institute for Virology in Johannesburg, South Africa, for sequencing. All viruses were type 1 and could be traced to common ancestral strains that circulate in disease-endemic reservoirs shared by northern Nigeria and southern Niger (WEAF-B genotype). The first importation occurred in Chad in August 2003 from northeastern Nigeria, and the outbreak spread to the adjacent countries of Cameroon in October 2003 and the CAR in December 2003.

In CAR, the first case occurred in a 19-month-old child living in Ndjoh village north of Bossembélé in Ombela M'Poko. A special mission by the World Health Organization/CAR officer determined that the child had not received OPV and had traveled 200 km into a northern region a few weeks before the onset date of December 16, 2003. The last case of acute flaccid paralysis in this region was noted on November 23, 1999. Supplemental immunization activities were conducted from March to April 2004. However, collected funds were not enough to cover the entire country, and only sanitary regions 1, 3, 4, 5, and 7 were included. The OPV coverage rate was estimated at 104% for the first round and 141% for the second round (CAR Ministry of Health, unpub. data). The second case occurred in April 2004 in Gadzi in sanitary region 2 in a 6-year-old nomad child who had not received OPV. The third case occurred in May 2004, in a village near where the second case was diagnosed, in a 23-month-old child who had not received OPV. Twenty-five other cases occurred between July and November in sanitary region 2 (Figure).

**Figure:**
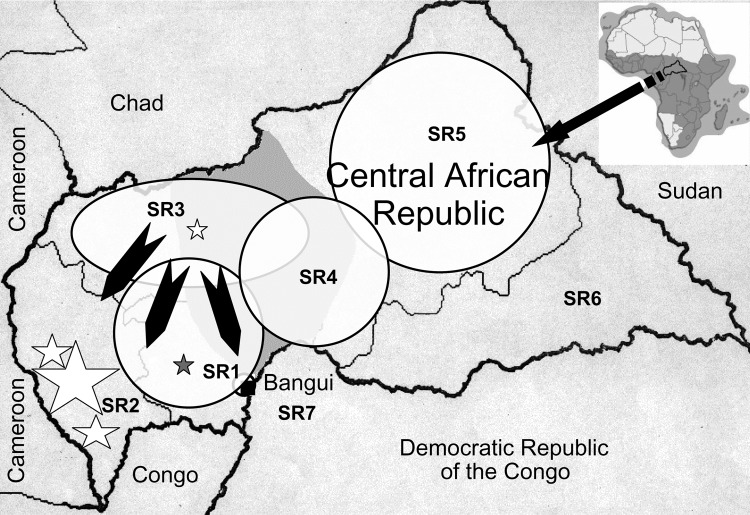
Supplementary immunization activities (SIA) areas, military conflicts, and migration movements, Central African Republic, 2001–2004. Gray star indicates first case of wild poliovirus type 1, Ombella-M'Poko (SR1), 2003; white stars indicate 2004 cases (1 in Ouham [SR3], 3 in Nana-Mambere [SR2], 3 in Sangha-Mbaere [SR2], and 23 in Mambere-Kadei [SR2]); circles indicate SIA areas, March–April 2004; arrows indicate 2001–2003 migration; dark gray shading indicates military conflict areas, 2001–2003.

This outbreak is the largest epidemic of WPV1 in CAR since July 2000, when the last case of WPV1 was isolated (*2*). Probable reestablishment of endemic poliovirus and possible diffusion of WPV1 to countries further south, such as the Democratic Republic of Congo, is a concern. Four main reasons may explain this outbreak: 1) the close links with countries, such as Chad and Cameroon, where WPV1 recently reemerged; 2) declining rates of routine vaccination and low population immunity after disruption of health service infrastructures and road networks; 3) displaced persons' living in crowded areas with little sanitation and poor water supply; and 4) lack of response preparedness to WPV importation. Epidemiologic investigation of the first case was not conducted until >1 month after onset and implementation of the polio immunization initiative in a limited area.

In May 2004, a decision was made by African Union health ministers to conduct a series of synchronized poliovirus campaigns across the African continent. Four rounds of National Immunization Days were conducted in CAR from August to December 2004. The OPV coverage rate in 600,000 children <5 years of age was estimated to be 89% in August, 98% in September, 102% in November, and 100% in December. Since November 2004, only 1 WPV1 case has been virologically confirmed in sanitary region 2. WPV1 has not been isolated in 2005.

To restore the gains made in polio eradication in Central Africa, WPV transmission must be interrupted in Nigeria and Niger (*3*). Until then, neighboring countries must implement high routine vaccination coverage and high-quality, supplemental immunization activities. In 2002, these steps successfully prevented importation of WPV into Bangladesh and Nepal during a resurgence of polio in India. Surveillance standards must also be maintained to ensure the rapid detection of any WPV importation, thus allowing timely response and containment.

## References

[1] Ministère de la Santé et de la Population de la République Centrafricaine. National Certification Committee. Annual report, 2003.

[2] Menard D, Gouandjika I, Mberio-Yaah F, Mokwapi F, Soro B, Djalai MI, Results of active surveillance of acute flaccid paralysis in the Central African Republic and Chad from 1995 to 2000. Med Trop (Mars). 2002;62:63–9. 12038182

[3] Centers for Disease Control and Prevention. Progress toward global poliomyelitis eradication, Nigeria, January 2003–March 2004. MMWR Morb Mortal Wkly Rep. 2004;53:343–6. 15123987

